# Subfield crop yields and temporal stability in thousands of US Midwest fields

**DOI:** 10.1007/s11119-021-09810-1

**Published:** 2021-05-08

**Authors:** Bernardo Maestrini, Bruno Basso

**Affiliations:** 1 Wageningen University & Research, Wageningen, The Netherlands; 2 Dept. Earth and Environmental Sciences, Michigan State University, East Lansing, USA; 3 W.K. Kellogg Biological Station, Michigan State University, Hickory Corners, USA

**Keywords:** Yield stability, Standard deviation, Two-way outlier, Big-data, Yield maps

## Abstract

Understanding subfield crop yields and temporal stability is critical to better manage crops. Several algorithms have proposed to study within-field temporal variability but they were mostly limited to few fields. In this study, a large dataset composed of 5520 yield maps from 768 fields provided by farmers was used to investigate the influence of subfield yield distribution skewness on temporal variability. The data are used to test two intuitive algorithms for mapping stability: one based on standard deviation and the second based on pixel ranking and percentiles. The analysis of yield monitor data indicates that yield distribution is asymmetric, and it tends to be negatively skewed (p < 0.05) for all of the four crops analyzed, meaning that low yielding areas are lower in frequency but cover a larger range of low values. The mean yield difference between the pixels classified as high-and-stable and the pixels classified as low-and-stable was 1.04 Mg ha^−1^ for maize, 0.39 Mg ha^−1^ for cotton, 0.34 Mg ha^−1^ for soybean, and 0.59 Mg ha^−1^ for wheat. The yield of the unstable zones was similar to the pixels classified as low-and-stable by the standard deviation algorithm, whereas the two-way outlier algorithm did not exhibit this bias. Furthermore, the increase in the number years of yield maps available induced a modest but significant increase in the certainty of stability classifications, and the proportion of unstable pixels increased with the precipitation heterogeneity between the years comprising the yield maps.

**Supplementary Information:**

The online version contains supplementary material available at 10.1007/s11119-021-09810-1.

## Introduction

Precision agriculture aims to maximize the efficiency of the inputs supplied to a crop (e.g. fertilizer, herbicide, irrigation, etc.) by defining zones to be managed homogenously within a field. The delineation of the homogeneous zones can be achieved either through the farming-by-soil approach, the farming-by-yield approach, or, more frequently, a combination of the two (e.g. Guastaferro et al., 2010; Taylor et al., 2007). The farming-by-soil approach uses mapped soil properties and farmers’ observations (Fraisse et al., 2001; Mzuku et al., 2005) to delineate zones in which crops respond homogenously to inputs. This approach has often been criticized because it does not consider soil-climate interaction (Basso et al., 2007; Basso & Antle, 2020). The farming-by-yield approach (Basso et al., 2007; Basso et al., 2016; Blackmore, 2000; Lark, 1998) instead uses yield as a proxy for soil variables, and historical yield maps to define management zones. The farming-by-yield approach allows for the consideration of temporal stability in management; for example, by dividing the field into zones that have high mean productivity (high-and-stable), zones that have low mean productivity (low-and-stable), and zones that are temporally unstable, such as zones that have high year-to-year yield variability (Basso et al., 2007, 2019; Blackmore, 2000; Maestrini & Basso, 2018; Martinez-Feria & Basso, 2020).

One of the main shortcomings in the current literature on management zone delineation is that studies tend to be focused on relatively few fields (e.g. Blackmore, 2000; Diacono et al., 2012; Gavioli et al., 2016; Taylor et al., 2007). There are exceptions, though, such as Stafford et al. (1999) who delineated the management zones of more than 60 fields in the United Kingdom using a fuzzy clustering approach. Also, field size, ranging typically between 4 and 12 ha, and the number of years of data available greatly limit the current possibilities for evaluating the effect of precision methods of a given set of delineated zones. Castrignanò et al. (2008) suggested that a minimum of thirty years of harvest data would be necessary to estimate temporal stability. However, a review of the literature shows that no study has investigated this claim, besides modeling studies. To evaluate weather larger datasets have the the potential to improve zone delineation, yield data were gathered from 768 fields located in eight states in the U.S., with up to 12 years of harvest data, covering a large diversity of topographies, soil types and climates. The crops present in this dataset are maize (*Zea Mays* L.), soybean (*Glycine max* Merr.), wheat (*Triticum* spp.), and cotton (*Gossypium* spp.).

Irrespective of the approach used to delineate zones, defining an algorithm that will effectively partition a field remains one of the main challenges for precision agriculture (Koshla et al., 2010). Creating an algorithm first requires the discretizating and clustering of one or more continuous mapped variables that may influence yield in various, possibly non-linear ways. Several approaches have been proposed in the literature, such as k-clustering (Fridgen et al., 2004), multivariate geo-statistical methods (Aggelopooulou et al., 2013; Castrignanò et al., 2000, 2008), and GIS layering (Kitchen et al., 1998). These approaches are powerful in their capacity to cluster high-dimensional datasets (i.e., including multiple variables), but they may not be easy to use because they do not offer a direct association of the classes to yield productivity or variability.

Here two farming-by-yield algorithms are presented to delineate homogenous management zones. These algorithms have the advantage of being relatively simple to implement and to interpret. The algorithm developed by Blackmore (2000) provided the bases of most algorithms available in the literature (e.g. Basso et al., 2007; Cox & Gerard, 2007). Blackmoore algorithm consists of two steps: first, the identification of the temporally unstable zones and second, the classification of the rest of the field based on their mean productivity. The two new proposed algorithms differ from Blackmore’s algorithm in the yield normalization and in the thresholds adopted. The first algorithm, *the standard-deviation algorithm*, uses the standard deviation of yield normalized across years as the measure of temporal stability. The second algorithm*, the two-way-outlier algorithm,* is non-parametric and is based on normalized yield ranks.

One of the main challenges of using of a farming-by-yield algorithm is the lack of unique criteria to establish a temporal stability threshold—as can be deduced from Table 1, which reports a variety of study criteria for defining temporal stability. An optimization procedure was used to find the thresholds that maximize the similarity of stability classification maps across different sets of years. The reasoning behind this optimization procedure is that any one set of data years available to a farmer is considered to be a sample of all possible years of harvest; Therefore the aim is to create an algorithm that maximizes the probability of having a similar management zone map if a different set of data years was available to the farmer.

**Table 1:** Review of the algorithms used in literature to define management zones based on yield

Authors	Size of fields and location	Crops	Years of yield data	Yield stability methods
Blackmore (2000)	6.7 ha (Bedforshire, UK)	wheat, rape	6 (1993–1998)	Yield normalization + classification based on mean and coefficient of variation
Reuter et al. (2005)	19 ha (Saxony, Germany)	spring barley, winter rye	3 (1999–2001)	Yield classified into 5 equally populated classes per year If class difference larger than 1 class, assigned 1, else 0, computing sum from all possible combinations
Basso et al. (2007)	8 ha (Rovigo, Italy)	corn, soybeans, wheat	5 (1998–2002)	Blackmore approach + study specific standard deviation threshold
Cox and Gerard (2007)	8.4 ha, 15 ha, 16 ha (Brooksville, MS, USA)	soybeans	4 (1998–2000 and 2002)	Blackmore approach + study specific spatial threshold (20%)
McEntee et al. (2016)	60 ha (Brookton, Australia)	wheat, pasture	4 (2009–2014, 2010 not used)	Blackmore approach + study specific standard deviation threshold (13%)
Yost et al. (2017)	36 ha (MO, USA)	corn, soybeans, wheat	22 (1993 to 2014)	Blackmore approach + study specific standard deviation threshold (25%)

A rigorous study of the temporal variability of yield must consider the shape of yield distribution, because skewed distributions may generate a correlation between mean productivity and temporal variability. Given its relevance for forecasting and insurance purposes, the skewness of yield distribution has been the subject of an intense debate since the mid-1960s (Day, 1965). More recent studies have shown that the distribution of yield is usually negatively skewed (long left tail in a normal distribution). For example, Joernsgaard and Halmoe (2003) observed a negative distribution for a variety of cereals in northern Europe, and Ramirez et al. (2003) observed a negative distribution for maize and soybean in the US Corn Belt. Several factors may influence yield skewness including nitrogen fertilization (Day, 1965), irrigation (Hennessy, 2009; Tack et al., 2012), and excess water or water deficit (Martinez-Feria & Basso, 2020). Maestrini & Basso, 2018 suggested that the unstable portions of the field are often characterized by a concave topography that facilitate stagnation in wet years and are wetter in dry years. Consequently, the hypothesis is that the proportion of unstable pixels is a function of site rainfall variability between seasons.

The overarching hypothesis of this study is that subfield yield distribution is skewed, which introduces a correlation between productivity and stability. The objectives of this research are to test this hypothesis and develop a new algorithm (the two-way-outlier) that is non-parametric (i.e., based on yield ranking) in order to disentangle unstable and low yield zones. The uncertainty of pixel classification and the influence of the number and variability of the data years available on the proportion of unstable pixels, is tested for the two algorithms.

## Materials and methods

### Data collection and processing

This study relies on yield data collected from 768 fields occupying a total area of 23,400 ha (Figure S2). The fields were located in eight states: Arkansas, Colorado, Illinois, Indiana, Iowa, Kansas, Michigan, and Missouri (Figure S1). The field sizes ranged from 0.85 ha to 212 ha and the mean field size was 30 ha. The crops investigated in this study were maize (1443 yield maps), soybean (724), cotton (261), and wheat (205). The details of the fields and yield maps by states and crops are reported in Table S1 and Table S2. The median number of years available per field was three, and the maximum was 12. Figure S2 shows the distribution of the number of years available per field. The farmers provided the data in various formats, depending on the yield monitor and software each farmer used. The yield data offer a variety of information about the harvesting process—most essentially, the dry yield volume of each crop. The raw files produced by the grain yield monitor were converted into shapefiles using Field Operations Data Model Viewer (FODM Viewer Version 3.11.09) and Spatial Management Software (SMS). Boundary calculation and rasterization operations were performed on the shapefiles using ArcGIS 10.4.1 and Python 2.7. The stability algorithms and the statistical analysis on the rasterized yield maps were implemented in R version 3.2, expanded by the package *raster* (Hijmans, 2016).

One yield map per field, year, and crop was derived and in any instance where two or more crops were farmed on one field in the same year, the map of that year was excluded from the dataset. Yield maps missing more than 25% of its data were also excluded. The analysis considered differences between cultivated crop species, but not crop variety.

The boundariess of each field were determined by merging all the point shapefiles and aggregating the points to polygons. From each crop dataset, the median was used to define a lower (0.1 × median) and higher (3 × median) boundary. All points below or above the boundary were treated as outliers and deleted. The points with the same longitude and latitude were dissolved to avoid duplicates. A spherical kriging model was applied with a pixel size of 2 by 2 m, a fixed radius with a distance of 20 m, and a minimum of 12 points. A final resolution of 30 × 30 m was obtained by clipping the temporary output to the boundary of the field and by smoothing it with a 10 × 10 pixel square by aggregating pixels. All the operations were performed on spatial objects (rasters, points, polygons) projected to the appropriate UTM zone and the meter was the unit of measure for the coordinates.

### Yield skewness and influence on stability

Yield skewness for every field-year yield map was calculated as follows:

$$\left( {\frac{{\mathop \sum \nolimits_{1}^{n} \left( {x_{i} - \overline{x}} \right)^{3} }}{n}/{ }\frac{{\mathop \sum \nolimits_{1}^{n} \left( {x_{i} - \overline{x}} \right)^{2} }}{n}} \right)^{3/2} ,$$

where *n* is the number of pixels present in the yield map and *x* is the vector of the pixel values of the field.

To investigate the influence of skewness on temporal variability, The correlation between mean and standard deviation (Fig. 2a) was tested and as well as correlation of yield to skewness observed in a field (Fig. 2b). The following steps were taken to test the correlation bewteen the mean and the standard deviation: (1) The fields with at least three years of data were selected to create a subset of the dataset, (2) Each field-year yield map was normalized (values were transformed to have µ = 0 and σ = 1), (3) For each pixel, the mean and the standard deviation of the values observed across the years were calculated, (4) The standard deviation was regressed against the mean using the following model

$$standard\, deviation = \alpha + \beta *mean + \varepsilon_{field} + \varepsilon_{res.}$$

where *ε*_*field*_ is a random effect of the intercept having as levels the individual field, and the *ε*_*res*_ are the model residuals.

To provide further evidence for the theory that negative skewness causes a negative correlation between the mean and the standard deviation, 10 independently and identically distributed variables (Figure S3 top panels) were tested, simulating negatively skewed, symmetrical (not skewed), and positively skewed variables. Each variable simulates the yield distribution observed in a field in a given year. A matrix of the type M[i, j] was generated where i represents the number of pixels in the simulated field and j represents the number of years. The mean and the standard deviation across the rows (i.e., across the years in this study’s analogy) were calculated and verified such that if the numbers are drawn from a negatively skewed distribution, the correlation between the mean and the standard deviation will be negative. Similarly, if the numbers are drawn from a positively skewed distribution, the correlation will be positive, and if the distribution is symmetrical there will be no correlation (Figure S3).

### Algorithms to define stability zones

Two algorithms to partition a field into three stability zones: unstable, low-and-stable, and high-and-stable are described and compared. The *standard-deviation algorithm* defines a pixel’s stability based on its standard deviation across years of normalized yield. The *two-way-outlier algorithm* defines stability based on variation in ranked yield values; this algorithm defines a pixel as unstable if at least one year’s yield was in the lowest 35% and another year was in the highest 35% of the yield distribution at field scale. The two algorithms employ two thresholds to divide high from low-and-stable from unstable, the values of these threshold have been defined so that they optimize the repeatibility of the management zones map. Further details on the definition of the threshold are given in the next section.

#### The standard deviation algorithm

The standard deviation algorithm partitions the pixel of a field into unstable, low-and-stable, and high-and-stable classes, based on normalized standard deviation and mean.

The algorithm is composed of the following steps:

1. Normalize (µ = 0, σ = 1) the values of each field-year yield map to allow for the comparison of yield maps for different years and crops.
2. For every pixel of the yield maps, calculate the mean and the standard deviation of the normalized yield across available years.
3. Define as unstable the pixels with a standard deviation greater than 0.8 and then divide the remaining pixels between low-and-stable (mean < 0.2) and high-and-stable (mean  ≥ 0.2).

#### The two-way outlier algorithm

The two-way outlier algorithm partitions the pixels of a field into the same three categories as the standard deviation algorithm, but defines unstable pixels as those pixels that jump from the bottom 35th to the top 35th percentile or viceversa. The steps of the algorithm are:

1. Normalize (µ = 0, σ = 1) the values of each field-year yield map to allow for the comparison of yield maps for different years and crops.
2. For each available year, divide the pixels into three categories: low (< 35% percentile), medium (points bounded in the interval between the 35th and the 65th percentile), and high yield (> 65th percentile).
3. If a pixel had at least 1 year in both the low and high categories, then categorize it as unstable.
4. Divide the remaining pixels based on mean normalized yield: classify them as low-and-stable if the mean is lower than 0.3, and high-and-stable if the mean is higher than 0.3.

#### Choosing the algorithm threshold through an optimization procedure

The thresholds to partition between high/low and stable-unstable were optimized to maximize the repeatability of the stability maps, i.e., the threshold that created the maximum probability of observing similar stability maps using different sets of years for the same field as inputs. To reach this goal, the fields were divided into several sets of calibration–validation years using different combinations. For example, for a field for which data from the years 2013 through 2018 were available, the threshold was optimized to ensure maximum agreement between a stability map for combined years 2013, 2014, and 2018, and a map for years 2015, 2016, and 2017. The optimization procedure—a simplex search procedure (Nelder & Mead, 1965)—was repeated 600 times, each time randomly selecting 30 fields (from the set of fields with at least 6 years of data). For each field, one combination of years was selected among all possible combinations. The 600 repetitions produced a bivariate distribution of 600 couples of optimum thresholds (one threshold for the standard deviation and one for the mean). For the standard deviation algorithm, the optimum threshold for normalized mean yield was 0.2, and the threshold for normalized yield standard deviation threshold was 0.75. The optimal thresholds for the two-way outlier algorithm were 0.3 (normalized mean yield) and 0.35 (i.e., a pixel must make a jump from being minor than the 35th percentile to be larger than the 65th percentile). The steps for the optimization of the threshold are in the supplementary information.

#### Uncertainty in the categorization of pixel and influence of weather

The patterns of a stability map depend on which years are used to produced it, the following is a procedure to identify pixels in the field with uncertain stability classification. The method is based on the partitioning of all yield maps (i.e., years of available data) for every field into two sets (set 1 and set 2) for every possible combination available for a field (i.e., all the possible ways to split the available years in calibration–validation sets). The goal of this analysis was to investigate the influence of the number of years of yield maps on the uncertainty of a stability map. The following steps were applied to each field having at least six years of data (129) to divide it into four categories, uncertain, unstable, low-and-stable, and high-and-stable:

1. For each combination of years, the stability maps were calculated using the two sets and for each pixel the categories obtained using the two sets were compared. A new map was created using the following rule to determine uncertainty: If a stability class defined using set A was equal to the stability class defined using set B, then the pixel was set as the stability class assigned by the sets. Otherwise, the pixel was set as *uncertain*.
2. After the exhaustion of all the possible combinations, the determination of the stability class of each pixel was done using a voting procedure, i.e., each pixel was assigned to the most frequent category (uncertain, unstable, low-and-stable, or high-and-stable) that occurred most frequently in that pixel across all the analyzed combinations.

#### Proportion of unstable pixels as a function of site rainfall variability between seasons

The dataset derived from these transformations was used to test the hypothesis that fields with yield maps from years in which summer precipitation differed had a larger proportion of unstable pixels. To test this hypothesis the stability maps and the meteorological data from the Daymet dataset corresponding to the years available (Thornton et al., 2017) were used. The proportion of pixels classified as unstable was modelled using the following linear mixed model:

$$logit\left( {\frac{{N_{unstable} }}{{N_{cells} }}} \right) = N_{years\, available} + \left( {rain_{wettest\, year} - rain_{driest\, year} } \right) + \varepsilon_{farmer} + \varepsilon_{res}$$

The variable *number of years available* was introduced because it is correlated with the proportion of unstable pixels as defined by the two-way outlier algorithm (Fig. 3). That is, with more years of data, there are more opportunities for a pixel to “jump” from the lowest 35th percentile to the highest 35th percentile. The term $\left( {rain_{wettest\, year} - rain_{driest\, year} } \right)$ represents the range of precipitation on a given field in the years with data available. The term $\varepsilon_{farmer}$ is a random effect of the number of farmers in the dataset as levels (number of levels 31), and $\varepsilon_{res}$ the residuals. The dataset comprised only the fields in rainfed states with at least six years of data.

#### Productivity, within-class variability and temporal variability of the three stability classes

For each crop and stability category the mean productivity over the years was derived by calculating the mean yield of each class in each stability map and by calculating the average for each combination of crop, stability class and year. The within-class variability of each class was calculated as the standard deviation of the pixels having the same stability category in the same stability map. The mean of these standard deviations by crop, year and stability was calculated and plotted over time (results are reported in Fig. 4).

The hypothesis that there were significant differences in temporal variability across stability classes (i.e., that the unstable pixels vary more between the years than the stable ones, Fig. 5) was tested using the following statistical model:

$$temporal variability = crop_{j} + stability class_{i,j} + \varepsilon_{state,j} + \varepsilon_{farmer,j} + \varepsilon_{residuals}$$

In the model, the *j* indices are the crops analyzed in this study (excluding cotton as explained below) and the *i* indices are the stability classes (low-and-stable, high-and-stable, unstable). The factors $\varepsilon_{state,j}$ and $\varepsilon_{farmer,j}$ are random effects taking as levels the states and the farmers (58 levels). The distribution of random effects $N\left( {0,\varepsilon } \right)$ was different for every crop.

Because the number of farmers growing cotton in the study dataset was limited the random effect $\varepsilon_{farmer,j}$ was dropped and the following simpler model was fit to the cotton data:

$$temporal\, variability = stability\, class_{i,j} + \varepsilon_{state} + \varepsilon_{field} + \varepsilon_{res}$$

For this model, the random effects were states and the number of fields (348 levels).

A Bonferroni-corrected posthoc test was applied to detect significant differences in the levels of the factor $stability\, class_{i,j}$ within each crop/algorithm between the temporal variability of the different stability classes.

The temporal variability models were fit on a dataset derived from the following steps which were followed using both stability algorithms separately:

1. The stability map for each field was calculated
2. For every crop planted at least twice, the standard deviation of the yield for every pixel across the years when the crop was cultivated was calculated.
3. A dataset was created where every pixel represents a row and the temporal variability, field ID, the stability class, the crop, the state, and the farmer ID are the columns.

### Statistical software

The skew of the distribution of each yield map was calculated using the R package *moments* (Komsta & Novomestky, 2015), and a skewed normal distribution was applied to each map using the package *sn* (Azzalini, 2017). The mixed linear models used to test a variety of null hypotheses were fit using the package *lme4* (Bates et al., 2015). The maximum likelihood of the multinomial models (optimization procedure and measure of classification certainty) was estimated using the *nnet* package (Venables & Ripley, 2002).

## Results

### Yield skewness and productivity of the unstable areas

**Fig. 1:**
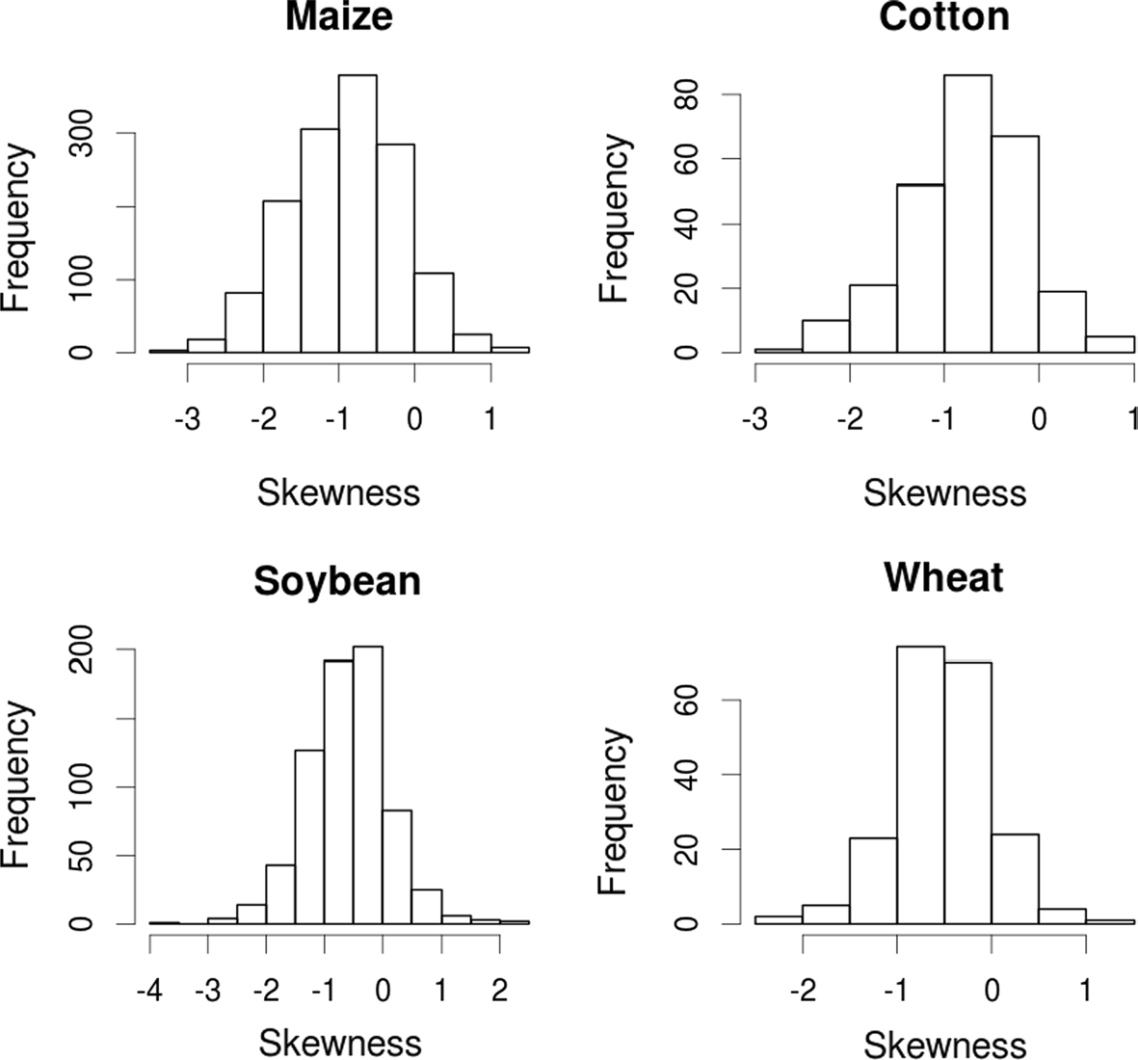
Distribution of yield skewness of different crops

**Fig. 2:**
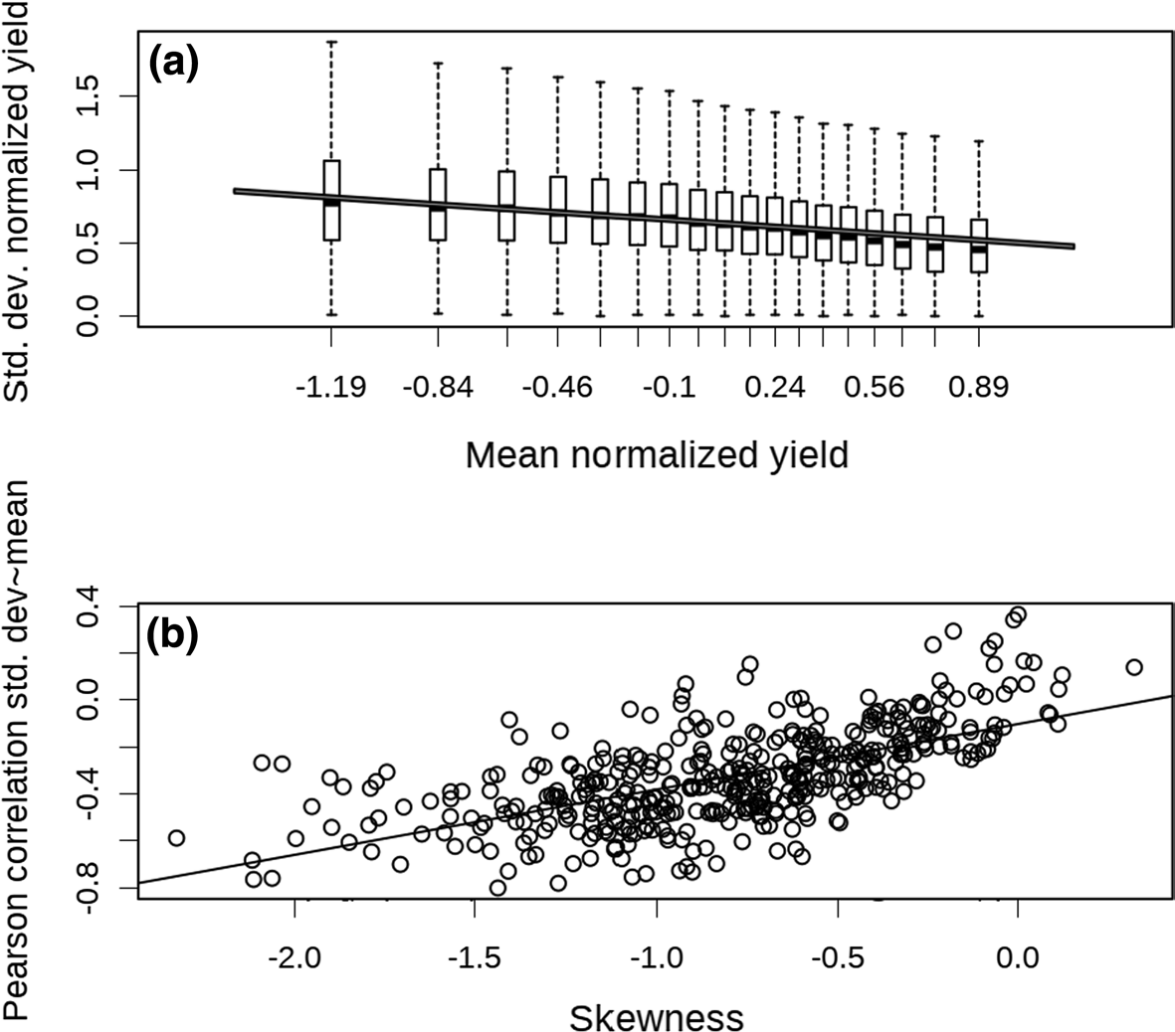
**a** Correlation between the mean and the standard deviation of the normalized annual yield. The analysis included only fields with at least three years of data. The barplots in this figure represent each pixel of each field binned by percentiles of 5% to facilitate visualization. The bold middle line represents the median of the bin, the bottom and top of the box the first and third quartiles and the two hinges are the 1.5 times the interquartile range of the data. The red line represents the regression line between the two variables. **b** Correlation between the mean skewness of within-field yield distribution and the negative mean-standard deviation correlation, for each field with more than three years of data

The majority of the yield distribution in the study was left-skewed (Fig. 1). The mean was negatively correlated with the standard deviation (both calculated across the years for normalized yield, p < 0.05, Fig. 2a). The correlation between the mean-standard deviation Pearson correlation coefficient and the mean skewness of the yield distribution of each field was positive (p < 0.05, Fig. 2b).

In the synthetic dataset drawn from a left-skewed distribution, the correlation between mean and standard deviation was negative, whereas in the right-skewed distribution the correlation was positive, and it was null if the data were drawn from a symmetric distribution (Figure S3).

### Stability classification and uncertainty in stability classification

The thresholds that maximized the repeatability of the standard deviation algorithm were 0.75 for temporal stability—measured as the standard deviation of the normalized yield over time—and 0.2 for mean normalized yield (Figure S4a). For the two-way outlier algorithm the repeatability was maximized by a threshold of 0.35 for temporal stability—meaning that a pixel was declared unstable if it fell for at least one year into the 65-100th yield percentile and at least one year in the 0-35th percentile—and 0.4 for mean normalized yield (Figure S4b).

On average 32% (σ = 11, Table 2) of a field was classified as unstable according to the standard deviation algorithm, whereas the two-way algorithm classified 41% (σ = 22, Table 2) of the pixels as unstable. The pixels that both algorithms classified as unstable represented 22% of the field. On average, 32% (9) of pixels were low-and-stable under the standard deviation algorithm, and 32% (12) under the two-way outlier approach. High-and-stable pixels represented 36% (11.6) of a field on average according to the standard deviation algorithm and 27% (11.3) on average according to the two-way-outlier algorithm (Table 2).

**Fig. 3:**
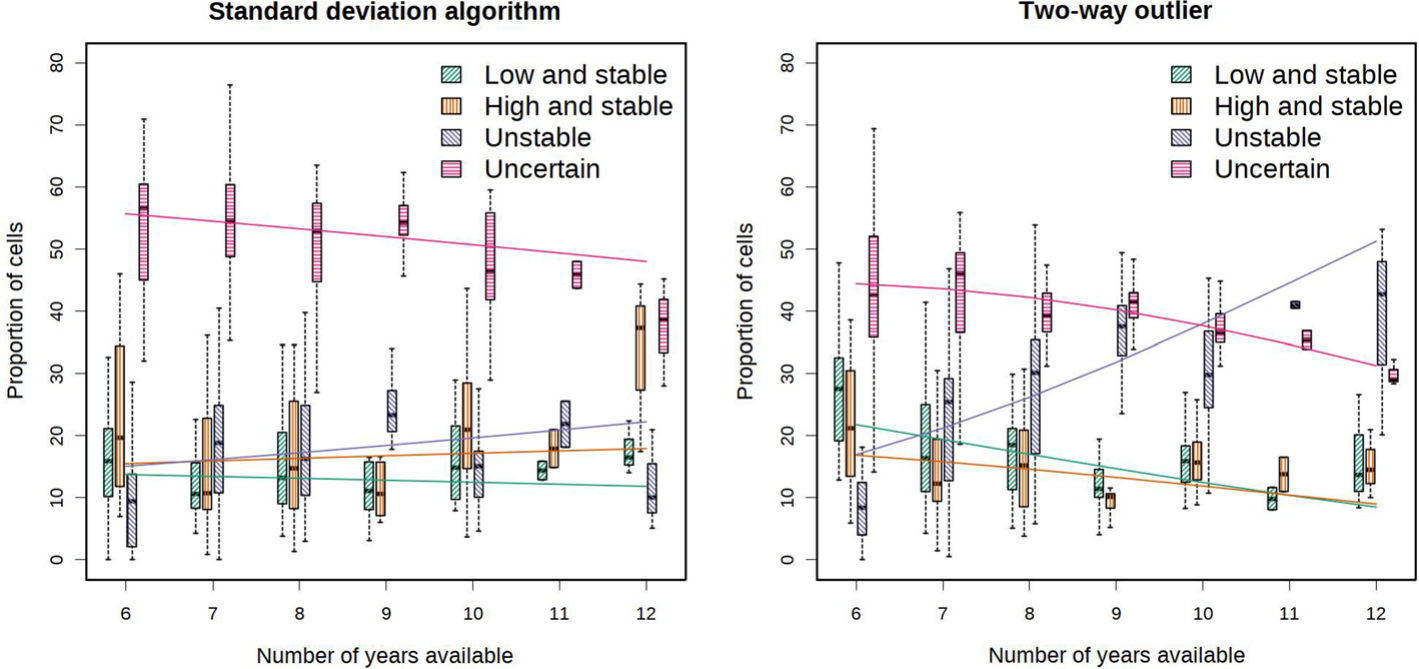
Influence of the number of years of data available for a field on the proportion of the field identified as low-and-stable (brown), high-and-stable (blue), uncertain (red), and on the portion of the field identified as unstable (orange). In the left panel, the standard deviation algorithm was used, whereas in the right panel the two-wayoutlier algorithm was used

Using the standard deviation algorithm, the increment in the number of years of yield available increased the number of unstable pixels mostly at the expense of the uncertain pixels, which dropped from 55 to 50% of the total when the number of years increased from six to 12 (p < 0.05, Fig. 3a). Using the two-way-outlier algorithm the proportion of unstable pixels increased with the number of years at the expense of all other categories (uncertain, low-and-stable and high-and-stable, p < 0.05, Fig. 3b). In fact, with six years of harvest data available, 15% of pixels were unstable, compared to 50% with twelve years of harvest data.

The proportion of unstable pixels increased with greater precipitation heterogeneity among the years available in the dataset. This observation held irrespective of the algorithm in use (Figure S5, p < 0.05). The analysis included only the fields located in traditionally rainfed states (Iowa, Michigan, Indiana, and Illinois) and with at least six years of data. Using the standard deviation algorithm, the fields with the largest heterogeneity in summer rain (300 mm of variation between available years of data) had on average (model prediction) 50% of pixels classified as unstable. The more uniform fields (100 mm) had 20% of pixels classified as unstable. Using the two-way outlier algorithm, the number of unstable pixels in fields with higher heterogeneity went up to 80%; in fields with more uniform precipitation, around 40% of pixels were unstable.

### Yield productivity, temporal variability, and within-class variability across stability classes

**Fig. 4:**
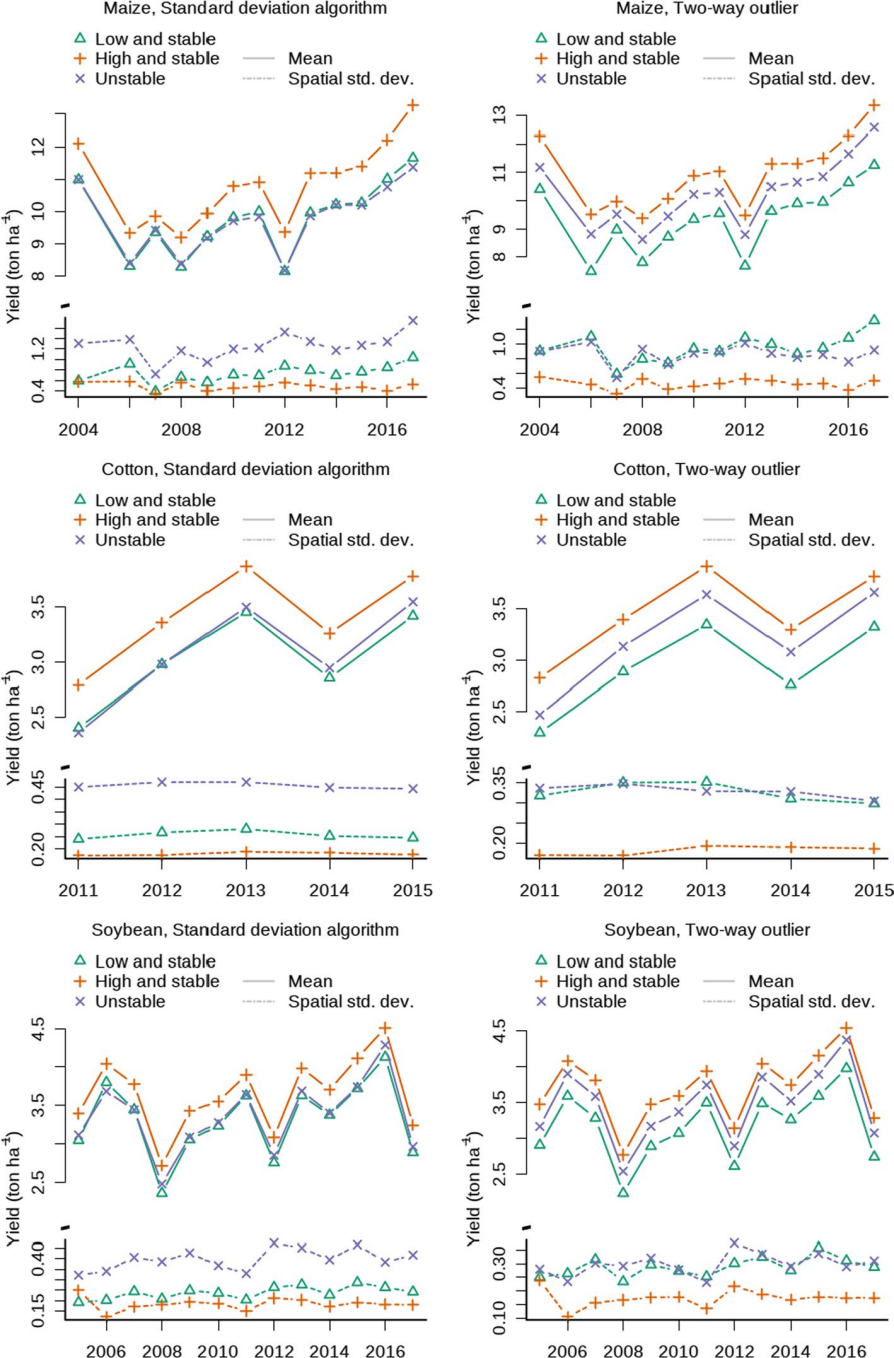

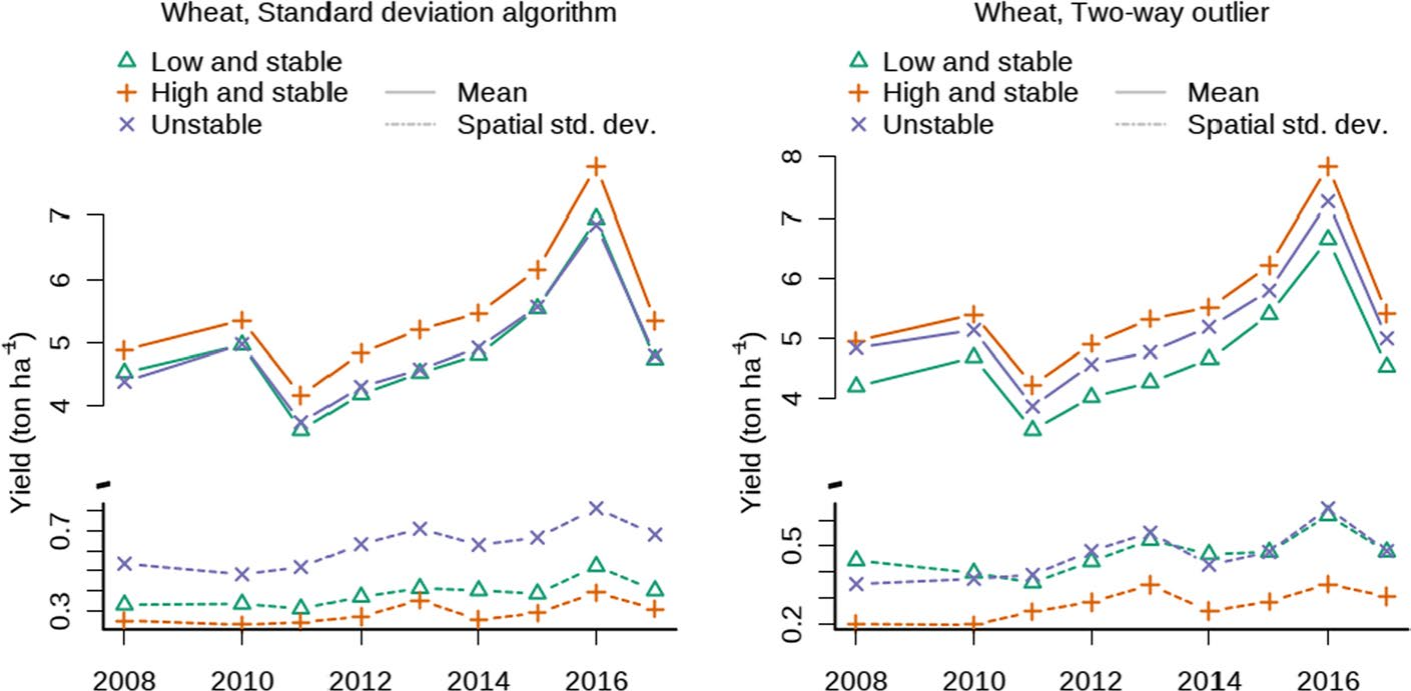
Yield (solid lines) and within-class variability (dotted lines) of the data aggregated by crop and year. The left panels represent the standard deviation algorithm, and the right panels represent the two-way outlier algorithm

For each crop, the yield averages of the low-and-stable and high-and-stable classes are reported in Fig. 4 (solid lines). Using the standard deviation algorithm, mean difference aggregated across the observed period between the high-and-stable and low-and-stable classes was 1.04 (σ = 0.18) Mg ha^−1^ for maize, 0.39 (σ = 0.02) Mg ha^−1^ for cotton, 0.34 (σ = 0.04) Mg ha^−1^ for soybean, and 0.59 (σ = 0.15) Mg ha^−1^ for wheat. Using the two-way-outlier algorithm, the mean difference between low-and-stable and high-and-stable classes was 1.62 (σ = 0.29) Mg ha^−1^ for maize, 0.53 (σ = 0.04) Mg ha^−1^ for cotton, 0.53 (σ = 0.05) Mg ha^−1^ for soybean, and 0.88 (σ = 0.16) Mg ha^−1^ for wheat.

The mean yield of the pixels classified as unstable by the standard deviation algorithm was similar to that of the low-and-stable pixels. In fact the differences—meand across the years—between low-and-stable and unstable were − 0.06 (σ = 0.12) Mg ha^−1^ for maize, 0.04 (σ = 0.07) Mg ha^−1^ for cotton, 0.05 (σ = 0.07) Mg ha^−1^ for soybean, and 0.03 (σ = 0.09) Mg ha^−1^ for wheat. In contrast, the two-way-outlier algorithm produced unstable pixels with an mean yield that was equally different from the low-and-stable and high-and-stable pixels (Fig. 4, continuous line). According to the two-way outlier approach, mean difference between the unstable and low-and-stable pixels was 0.90 (σ = 0.23) Mg ha^−1^ for maize, 0.27 (σ = 0.07) Mg ha^−1^ for cotton, 0.30 (σ = 0.04) Mg ha^−1^ for soybean, and 0.51 (σ = 0.51) Mg ha^−1^ for wheat.

Unstable pixels had the highest within-class variability—i.e., the standard deviation of the yield of pixels with the same stability class in each stability map—followed by low-and-stable pixels and then high-and-stable (Fig. 4, dashed lines).

**Fig. 5:**
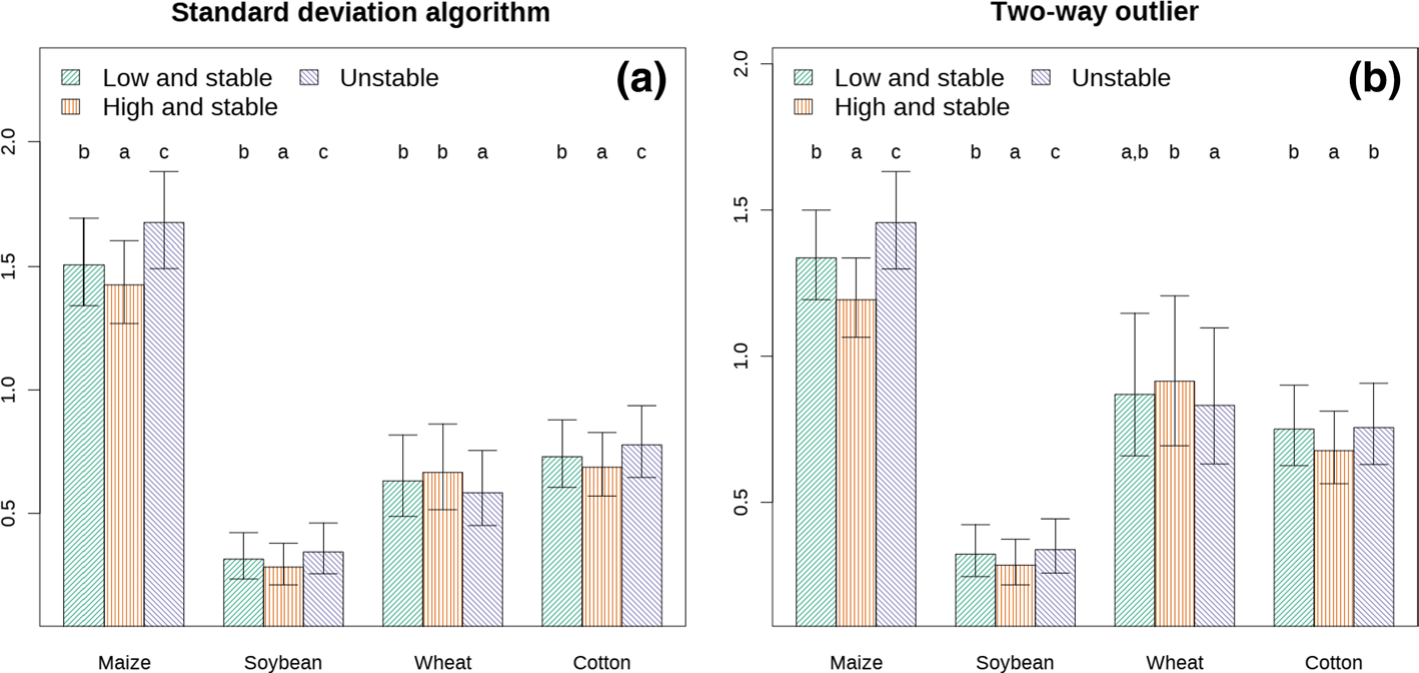
Mean temporal variability by crop (expressed as standard deviation of year-to-year yield). In the left panel, the standard deviation algorithm was used, whereas in the right panel the two-way outlier algorithm was used. The letter indicates significant differences between stability classes within the same crop

Using the standard deviation algorithm, the unstable pixels were between 14 (cotton) and 20% (soybean) more variable that the high-and-stable pixels, whereas the two-way outlier algorithm found differences ranging from 11 (cotton) to 21% (soybean). The temporal variability of the unstable pixels differed less sharply from the low-and-stable pixels. The differences between the temporal variability of the unstable pixels and the low-and-stable pixels ranged between 7 (cotton) and 13% (maize) for the standard deviation algorithm and between 1.3 (cotton) and 9% (maize) for the two-way outlier algorithm. Using the standard deviation algorithm, the temporal variability of wheat was higher in high-and-stable pixels than unstable pixels (p < 0.05 using posthoc test, Fig. 5), and no significant difference was observed under the two-way outlier algorithm.

## Discussion

### Asymmetric yield distribution causes a positive correlation between productivity and temporal stability

The analysis of the yield distribution of 5520 field yield maps revealed that within-field yield distribution was negatively skewed, as indicated also by a growing body of literature (see the review by Hennessy, 2009 and Ramirez et al., 2003 in contrast to Day, 1965).

The analysis of the data confirmed the hypothesis that negatively skewed yield distribution causes a negative correlation between the mean and the standard deviation of normalized yield measured across the available years of data. Two independent analyses supporting the hypothesis are provided, one from analysis of the study dataset, and one from synthetic data. The negative correlation between productivity and variability imply that the less productive areas are also more unstable, and therefore even more critical for the financial sustainability of a farm operation.

### The standard deviation algorithm and the two-way outlier algorithm

The negative correlation between mean productivity and temporal variability brought us to two possible interpretations of yield stability, implemented through two different algorithms. The standard deviation algorithm uses the standard deviation of the normalized yield to identify unstable pixels, and the two-way outlier algorithm identifies as unstable the pixels that oscillate between high and low yield. The first interpretation has important practical implications for management because it helps to identify the areas that show the highest variation and therefore contribute most to the volatility of farm profits. However, because of the negative correlation between productivity and temporal variability, some of the pixels classified as unstable by the standard deviation algorithm are unstable because of the inherent highly random variability typical of the low-yielding pixels. Approximately two-thirds of pixels unstable based on the standard deviation algorithm were also categorized as unstable according to the two-way-outlier algorithm (Table 2). The two-way-outlier method identifies as unstable the points that “jump” between relatively low and relatively high production. The erratic behavior of this type of unstable pixel is more likely dependent on climate-soil interaction. For example, it is possible that the pixels classified as unstable suffer from heavy rainfall events during crop establishment and perform relatively better in dry summers (Maestrini & Basso, 2018). These causes of instability may be addressed for example by choosing ad-hoc sowing rates, ensuring adequate drainage or using flood resistant genotypes (Martinez-Feria & Basso, 2020).

### More data years moderately decreases the uncertainty of a stability map

Areas characterized as uncertain have a substantial lack of confidence about their classification; i.e., their classification cannot be consistently repeated if a map for the same field was produced using yield data from different years. Uncertainty in pixel classification, which is measured as repeatability of a map using a calibration–validation dataset split approach, decreased with the number of years of data available (Fig. 3), in accordance with the principle that the uncertainty of an estimate decreases with more replicates available. Nonetheless, the improvements in map repeatability derived from having more years available were relatively modest. In fact using the standard deviation approach passing from 6 to 11 years of data decreased the share of uncertain pixels from 55 to 50% on average (p < 0.05, Fig. 3). Increasing the number of available years decreased the number of uncertain pixels but sharply increased the number of unstable pixels, suggesting that estimates of temporal stability are more uncertain than estimates of mean production.

### The number of years and their rainfall variability influence the proportion of unstable pixels

In the two-way outlier approach, there was a sharp increase in the proportion of unstable pixels when more years of data were available (Fig. 3), because the number of years clearly influences the chances that a pixel “jumps” from one tier of production to another (e.g. from low to high). Undeniably, some of the these “jumps” are the result of unexplained stochastic variability. However, an increase in the number of data years increases the possibility of encountering years with extreme climatic conditions (e.g. very dry) that will reveal the correct classification of some potentially unstable pixels. In fact, a peculiar trait of the unstable pixels is that their relative performance (i.e., their yield relative to the rest of the field) is strongly driven by climate-soil interaction, whereas the relative performance of the stable pixels does not depend on climate. From this assumption, it follows that if the weather was relatively uniform in the years with data available to build a stability map, the year-to-year behavior of unstable pixels will reflect that weather uniformity. Therefore, some of the pixels that are potentially unstable (i.e., those pixels whose relative performance is determined by soil-climate interaction) may not be identified as unstable if the weather is uniform. This thesis is confirmed also by the positive correlation between the heterogeneity of cumulative rain in July and August (a proxy for climate) and the proportion of unstable pixels (Figure S5). This suggests that a stability map can only reflect the weather variability of the years that build the map, and that having years that exhibit large differences in weather will help to reveal the true temporal stability of pixels.

### Mean, temporal variability, and within-class variability of yield across stability zones

The mean difference between the yield in high-and-stable zones and in low-and-stable zones was approximately 1 t ha^−1^ for maize and wheat and 0.5 t ha^−1^ for soybean and cotton (Fig. 4). According to the standard deviation algorithm, the production from unstable field pixels was similar to low-yield pixels because of the positive correlation between productivity and temporal variability. On the contrary, the two-way outlier algorithm found that the mean yield of unstable zones was between the mean production of the high and low stable pixels (Fig. 4). This suggests that the two-way outlier algorithm corrects the standard deviation algorithm’s bias toward the low-and-stable mean yield.

The most distinctive characteristic of the unstable areas is their temporal variability. The unstable areas identified through the standard deviation algorithm have greater temporal variability than stable areas. This was true for all unstable areas, for all crops except wheat (Fig. 5). The difference could stem from the fact that the growth cycle of wheat differs substantially from the other crops and/or that wheat was less represented in the study dataset. In fact, only 10% of fields had at least two wheat maps. Therefore the estimate for wheat variability derives mostly from maps created for other crops. Unstable areas identified by the two-way outlier algorithm show greater temporal variability only in the case of maize and soybean (Fig. 5). This suggests either that stability maps created using multiple crops have limited predictive power for a single crop, and/or that temporal variability estimated using percentiles differs substantially from variability evaluated with statistics based on distribution parameters such as standard deviation.

The within-class variability, measured as the standard deviation of the normalized yield of the pixels belonging to the same stability class and yield map, of unstable zones was higher than that (Fig. 4) of stable zones because stable pixels are by definition more homogenous than unstable pixels as their range is restricted, compared to unstable pixels that are not bounded to a mean (their normalized mean needs to be larger than 0.2).

## Conclusion

The distribution of within-field yield is negatively skewed, which causes temporal instability in low-yield pixels. This needs to be considered when using the farming-by-yield approach. A rank-based classification algorithm (two-way-outlier) can be used to discern between low and unstable pixels, however, they suffer from a strong dependence on the number of years available, and the weather. This indicates that in order to capture the yield variability it is important to observe a field for many and different years. Therefore it is important to rely on a standard platform for the long-term storage of the data.

A method to define the thresholds and uncertainty in pixels classification was proposed in this study, however it is clear that there is a certain level of subjectivity in the creation of the stability maps. Therefore it is important that decision support systems show the uncertainty in classification and the implications of choosing one algorithm over another one.

**Table 2:** Contingency table of the classification according to the two-way-outlier algorithm and the standard -deviation algorithm expressed as percent of the number of pixels

	Low-and-stable Two-way outlier	High-and-stable Two-way outlier	Unstable Two-way outlier	Sum
Low-and-stable Standard deviation algorithm	22.2 (11)	0 (0)	10.2 (6.3)	32.4 (8.5)
High-and-stable Standard deviation algorithm	1.8 (1.5)	26.1 (11.9)	8.1 (6.2)	36 (11.6)
Unstable Standard deviation algorithm	7.5 (3.8)	1.8 (2.4)	22.3 (15.5)	31.6 (16.5)
Sum	31.5 (12)	27.9 (11.3)	40.5 (22.2)	100 (0)

A contingency was calculated for each field. For each pixel of the contingency table, the mean and the standard deviation (n = 428) was calculated across the contingency table of all the fields. The number in parenthesis is the standard deviation

## Supplementary Information

Below is the link to the electronic supplementary material.

Supplementary file1 (DOCX 637 kb)
