# Supplementary material for: Subfield crop yields and temporal stability in thousands of US Midwest fields
Source: Precis Agric. 2021 May 8;22(6):1749–67. doi: 10.1007/s11119-021-09810-1 (PMC8553677; doi:10.1007/s11119-021-09810-1)
Supplement: Supplementary file 1 — Supplementary file1 (DOCX 637 kb) [file 11119_2021_9810_MOESM1_ESM.docx]

Supplementary information

**Skewness of the distribution of within-field crop yield in the US Midwest and its implication for the delineation of management zones and its uncertainty**

Bernardo Maestrini^1,2^ and Bruno Basso ^2,3^

- - - 1. Wageningen University & Research

1. Dept. Earth and Environmental Sciences, Michigan State University
2. W.K. Kellogg Biological Station, Michigan State University


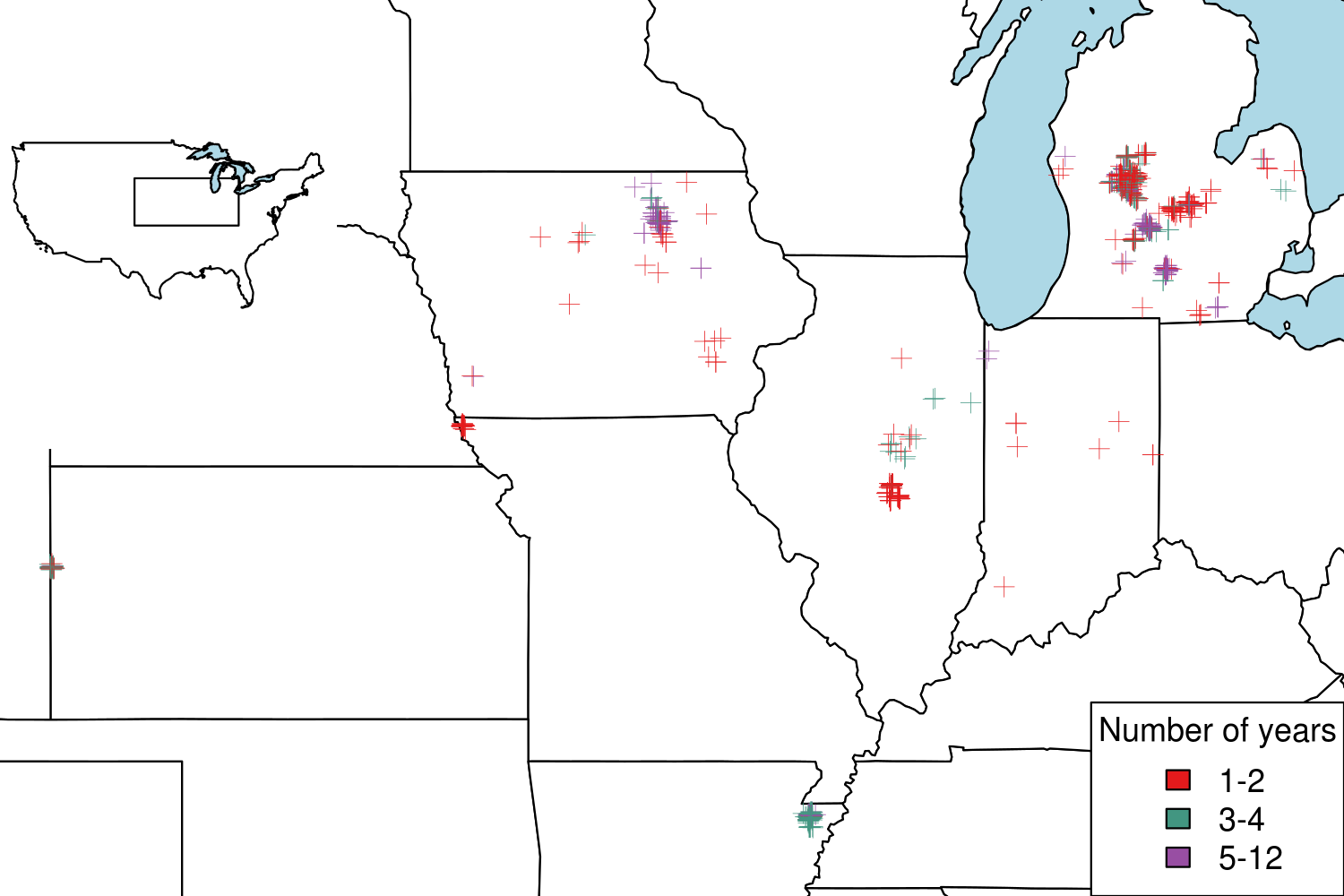


Figure S 1: Location of the 768 fields analyzed in this study.

Table S 1: Number of yield maps by crop and state.

|  | *Arkansas* | *Colorado* | *Illinois* | *Indiana* | *Iowa* | *Kansas* | *Michigan* | *Missouri* | *Sum* |
| --- | --- | --- | --- | --- | --- | --- | --- | --- | --- |
| Maize | 67 | 19 | 66 | 20 | 306 | 51 | 894 | 20 | 1443 |
| Cotton | 261 | 0 | 0 | 0 | 0 | 0 | 0 | 0 | 261 |
| Soybean | 119 | 0 | 42 | 9 | 117 | 0 | 422 | 15 | 724 |
| Wheat | 0 | 11 | 0 | 0 | 0 | 32 | 162 | 0 | 205 |
| Sum | 447 | 30 | 108 | 29 | 423 | 83 | 1478 | 35 | 2633 |

Table S 2: Number of fields by state.

| *Arkansas* | *Colorado* | *Illinois* | *Indiana* | *Iowa* | *Kansas* | *Michigan* | *Missouri* | *Sum* |
| --- | --- | --- | --- | --- | --- | --- | --- | --- |
| 119 | 8 | 79 | 10 | 82 | 29 | 420 | 21 | 768 |


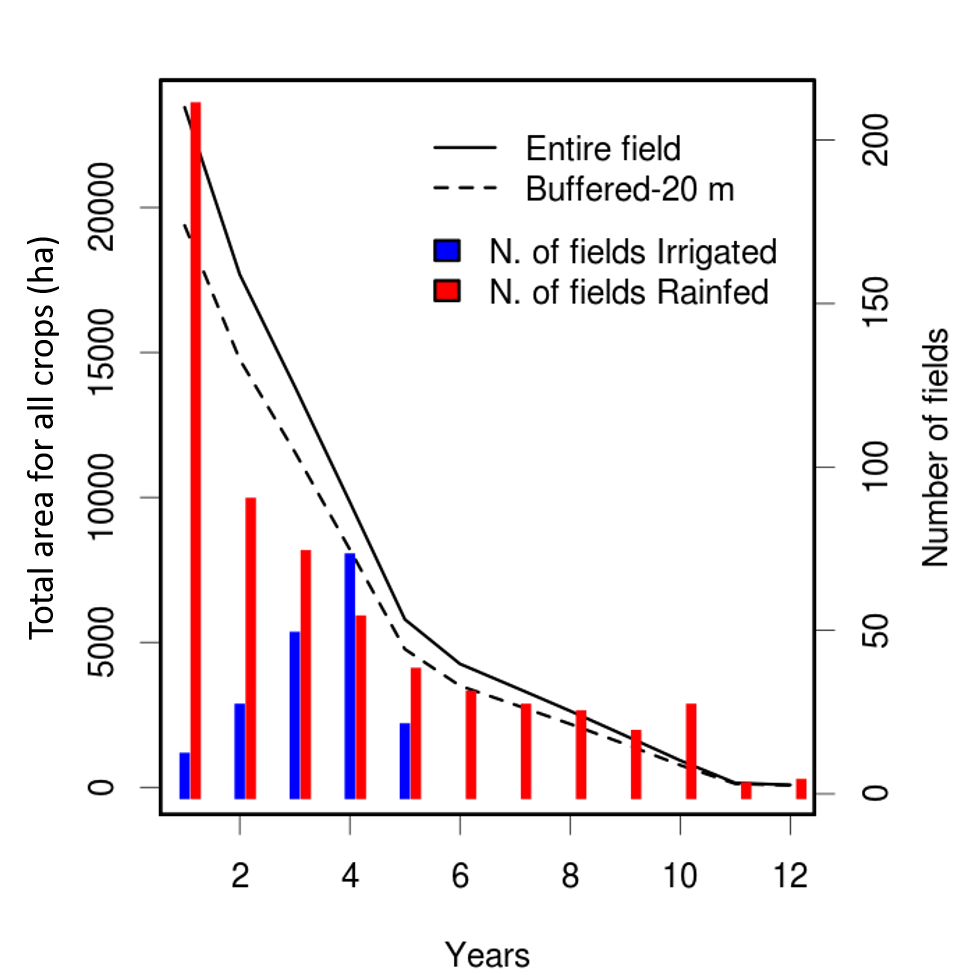


Figure S 2: Plot of the total area of the fields and the number of fields analyzed in this study as a function of the number of years of yield maps available. The solid line (left axis) refers to the full area of the fields, the dashed line the total area of the fields minus a buffer of 20 m from the edges, the blue and red bars (right axis) refer to the number of irrigated and rainfed fields available for a given number of years.


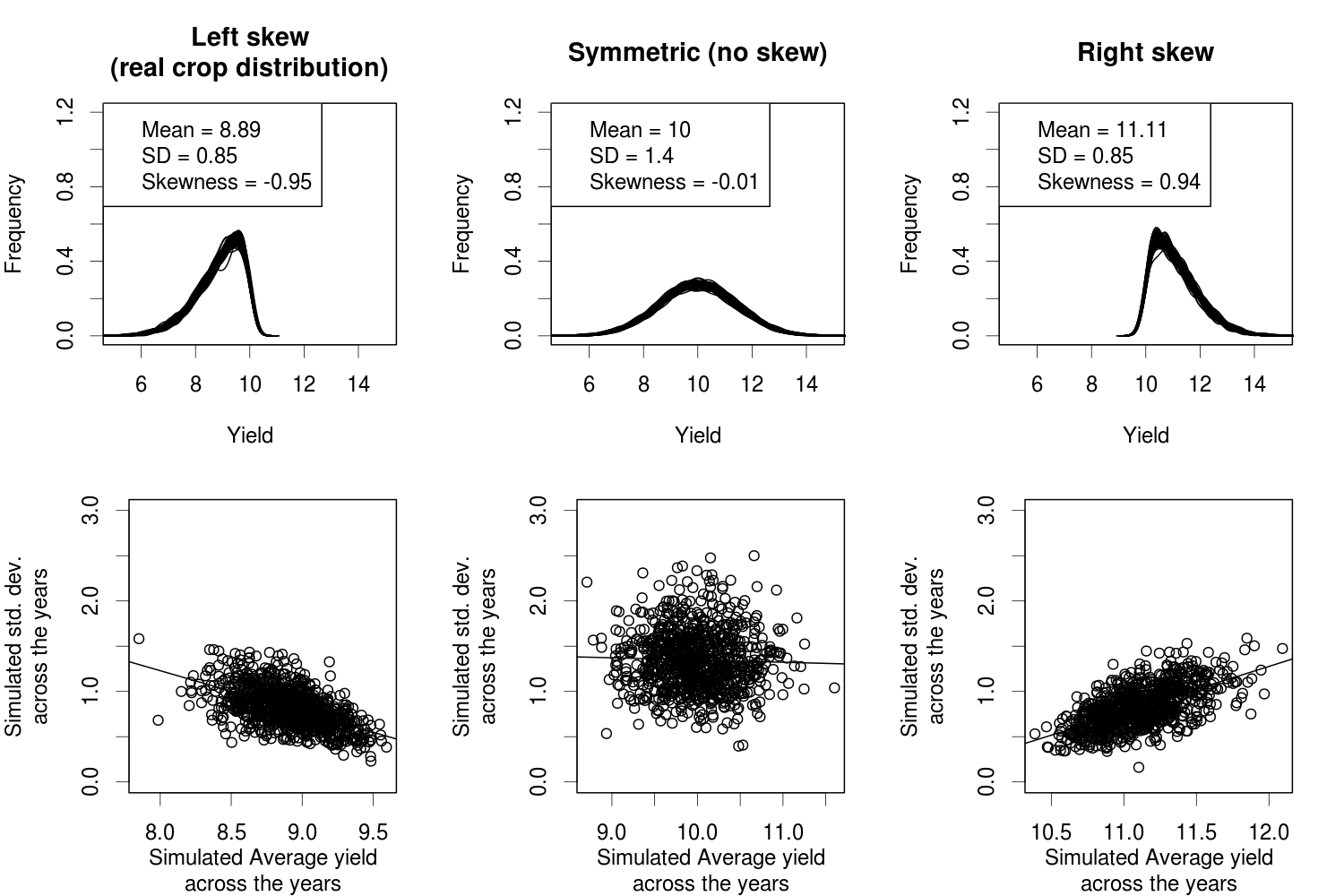


Figure S 3: Simulation of 10 independently and identically distributed variables demonstrating left skew, symmetry (not skewed), and right-skew (top panels). Each variable simulates the yield distribution observed in a field in a given year. The bottom panels show the correlation between the multi-year averages and the sd. This simple simulation shows that the skewness of the variables influences the correlation between the multi-year averages and the standard deviations.


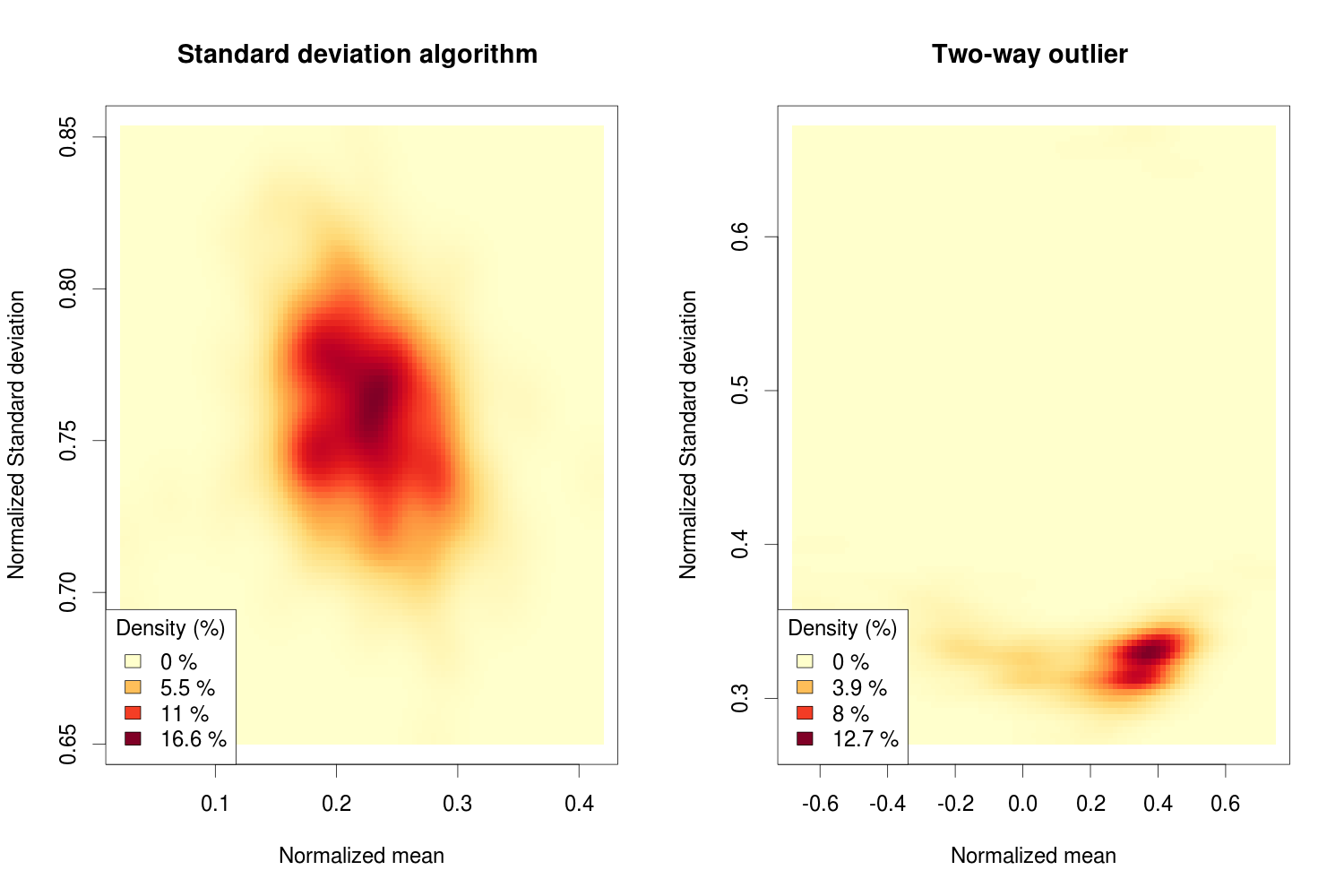


Figure S 4: Optimization of the parameters in the standard deviation algorithm (left) and two-way outlier algorithm (right). We optimized the parameters to maximize the repeatability of the stability maps. We visually identified the best threshold for the parameters as 0.2 for the mean and 0.75 for the standard deviation in the standard deviation algorithm, and 0.15 for the mean and 0.35 for the standard deviation in the two-way-outlier algorithm.


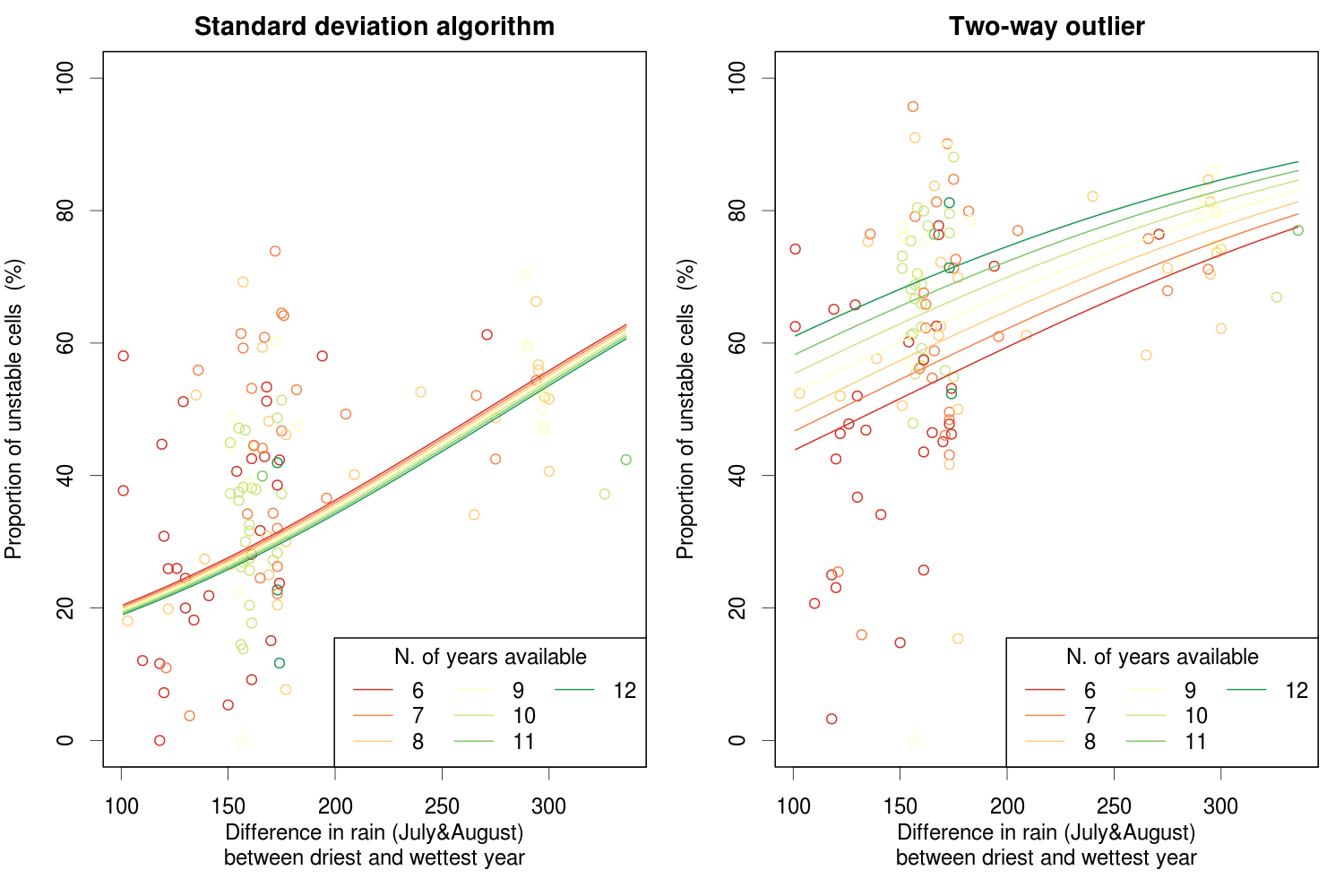


Figure S 5: Influence of observed rain variability and of the number of years of data available on the proportion of pixels categorized as unstable, using the standard deviation algorithm and the two-way outlier algorithm. The dataset was limited to fields with at least 6 years of data.

## Algorithm for the definition of the optimum thresholds

For each of the 600 repetitions, we used the following algorithm to identify the optimal threshold:

1. Randomly select 30 fields from those with at least 6 years of yield maps (109).
2. Split the years available for each field into a calibration and a validation dataset. If there was an odd number of years available, the calibration dataset was composed of one more year than the validation dataset (e.g. if seven years were available the calibration set was composed of 4 years and the validation of 3).
3. Start of the optimization procedure using the Nelder and Mead (Nelder and Mead, 1965) optimization procedure (default in R *optim* function):
   1. Select one starting value for each of the two thresholds from a uniform distribution. For the standard deviation algorithm, the starting values were chosen from a uniform distribution in the domain [0.5, 1.5] for the temporal variability threshold (that being a standard deviation cannot be smaller than 0) and in the domain [-1, 1] for average productivity. For the two-way outlier algorithm, the domains were [0.05, 0.45] for the percentile threshold (the parameter governing the definition of instability) and [-1, 1] for average productivity.
   2. For each subset (calibration or validation) of the 30 randomly selected fields, we derived the stability map using the chosen threshold. To define the optimal threshold for the standard deviation algorithm, we used the standard deviation algorithm to calculate the stability maps, whereas for the optimization of the two-way outlier we used the two-way outlier algorithm.
   3. We calculated the maximum likelihood of a multinomial model in which the response variable was the stability map derived from the validation set and the predictor was the stability map derived from the calibration set. The objective function maximized by our optimization procedure was the maximum likelihood for the model.
4. Record the optimum parameters obtained using the field subset.

After we obtained 600 couples of optimum parameters, we used a bivariate density plot to visually identify the area characterized by the highest density and therefore identify the threshold values that ensure the maximum repeatability of the stability maps (Figure S 10).
